# Participatory and Transdisciplinary Studies of *Brucella* Infection in Humans and Animals in Yunnan Province, China—Lessons Learned

**DOI:** 10.3390/tropicalmed6030134

**Published:** 2021-07-15

**Authors:** Wengui Li, Xiangdong Yang, Johanna F. Lindahl, Guorong Yang, Jeffrey Gilbert, Fred Unger

**Affiliations:** 1 College of Veterinary Medicine, Yunnan Agricultural University, Kunming 650201, China; wenguili@yeah.net; 2 Yunnan Institute of Endemic Disease Control and Prevention, Dali 671000, China; xdyang877@163.com; 3 Department of Biosciences, International Livestock Research Institute, Nairobi 30709, Kenya; 4 Department of Medical Biochemistry and Microbiology, Uppsala University, 75123 Uppsala, Sweden; 5 Department of Clinical Sciences, Swedish University of Agricultural Sciences, 75007 Uppsala, Sweden; 6 Yunnan Academy of Grassland and Animal Science, Kunming 650212, China; 7 Food and Agriculture Organization, 00153 Rome, Italy; jeffrey.gilbert@fao.org; 8 International Livestock Research Institute, Hanoi 100000, Vietnam; f.unger@cgiar.org

**Keywords:** brucellosis, ecohealth, zoonotic disease, behavior change, disease transmission, One Health

## Abstract

Brucellosis is an important zoonosis occurring globally. In addition to the risk for disease in humans, the disease causes production losses, since the disease in livestock is characterized by abortion and other reproductive failures. The disease is a public health concern in China, but no information is available on knowledge, perception and awareness of potential risk groups such as farmers, butchers and animal health workers; yet successful control requires compliance of those affected groups to be effective. Following the principles of the Ecohealth approach, emphasis was given to participation of all relevant stakeholders, use of qualitative and quantitative tools, and cross-sectorial collaboration. Data collection included on-farm questionnaires (N = 192) and collection of bulk milk samples of goat (N = 40), cattle (N = 45) and buffalo (N = 41) from farms, as well as serum samples (N = 228) from humans. Milk samples were tested with an ELISA for presence of antibodies, while a serum agglutination test was used for human samples. Qualitative work included 17 focus group discussion (FGD) with villagers and 47 in-depth interviews (IDI) with village animal health workers, doctors, and butchers, focused on knowledge, perception and awareness on zoonoses including brucellosis. Results from questionnaires indicate that abortions are a common problem; cattle with abortion history are kept for further insemination and the milk still consumed or sold. Antibodies against *Brucella* were detected in cows’ (5/45) and goats’ (1/40) milk samples, and in human samples (5/126) in Yiliang, while in Mangshi, all buffalo (N = 41) and humans (N = 102) were negative. FGD and IDI results showed an alarmingly low knowledge and awareness on zoonoses; particularly, low awareness about brucellosis was noted, even among the professional groups. Collaboration between village animal health workers and doctors was uncommon. No confirmed brucellosis cases were found in retrospective investigation of hospital and veterinary stations. This study demonstrates the presence of brucellosis in livestock and humans in Yunnan, indicating a non-negligible risk for humans. It is also made apparent that there is a need for increased awareness among both farmers and professionals in order to reduce the risk of zoonotic transmissions.

## 1. Introduction

Many zoonotic diseases cause a double burden for farmers, since they are not only causing disease and suffering in humans, often with decreased income as a consequence, but they are also causing disease in livestock, with reduced productivity, and further reduced incomes. Of around 1500 pathogens that affect humans, around 200 human diseases are classified as emerging infectious diseases (EID), and 75% of these are zoonotic [1]. Southeastern Asia is a major focus for emerging and re-emerging diseases, a hot spot with 42% of the world’s EIDs, including severe acute respiratory syndrome (SARS) and avian influenza (AI), in the past [2].

While EIDs are getting much international attention, endemic diseases are often more neglected. Brucellosis is a significant and notifiable zoonotic disease across the world, including China, where the number of reported cases has increased each year from the beginning of the 21st century, and indeed was among the top 10 notifiable infectious diseases during 2000–2006 [3].

In China, brucellosis was first reported in 1905 and historically was highly prevalent in both humans and domestic animals. During the 1950s to the 1970s, annual incidence was over 1 per 100,000 population in both humans and livestock. After application of strategic prevention and control measures (including vaccination), its prevalence decreased profoundly. In the 1980s and 1990s, annual incidence has dropped to 0.2 per 100,000 population, both in humans and livestock [4]. However, annual reported cases of human brucellosis has showed an upward trend since 2000 [5]. In 2009, 35,816 brucellosis cases were reported. The annual incidence was 2.7 per 100,000 population [6].

In Yunnan Province, brucellosis was first reported in 1962 [7]. Cases were increasing over time from 2010 to 2015, making brucellosis a potentially re-emerging infectious disease [8]. According to national surveillance data, increasing human brucellosis cases in Yunnan Province was concurrent with an increasing trend in animals [9,10]. The current control strategy for brucellosis in China consists of a surveillance and slaughter policy; in addition vaccination is applied, but limited to high prevalence areas [11]. A successful control of brucellosis would require involvement of various stakeholders to be effective. In particular this should include the compliance of farmers which are most affected not only by the diseases, but also by the control measures. For these reasons, in this study four institutions—representing researchers from animal science, veterinary medicine and public health—were inspired to work together to demonstrate that transdisciplinary research can contribute to a better understanding of zoonoses, selecting brucellosis as the pilot disease through a collaborative process. The project objectives included to understand perception, awareness and behavior of stakeholders, including potential risk groups, towards brucellosis, to investigate the impact of these diseases in pilot communities and hospitals, and to demonstrate how an integrated approach can help to improve collaboration between different stakeholders and contribute to a better control.

## 2. Materials and Methods

The planning and fieldwork were conducted between December 2010 and May 2013.

### 2.1. Study Sites

**Figure 1:**
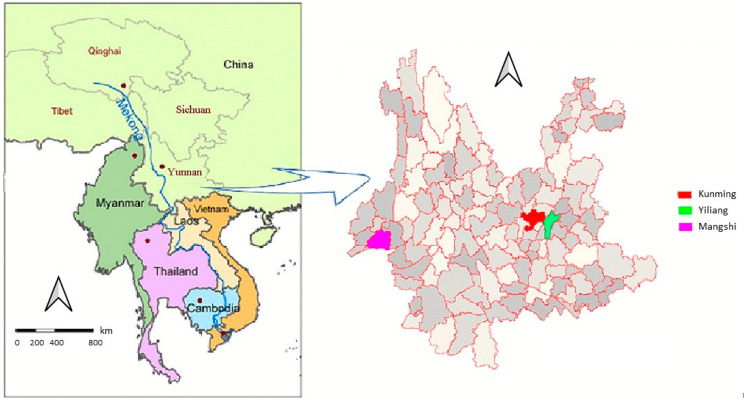
Location of Yiliang county and Mangshi county level city in Yunnan province of China.

Mangshi County level city and Yiliang County in Yunnan Province were selected purposefully as study sites based on their geographical, socio-cultural and economic differences (Figure 1). Yiliang County, with an area of 1880 km^2^, is a peri-urban area of the provincial capital Kunming, with an elevation of 1540 m and a north subtropical monsoon climate. Yiliang has a population of 394,950. Most of the population is ethnic Han (the largest ethnic group in China) and most dairy ruminants are cattle and goats. Mangshi County level city is located in western Yunnan province with an area of 2901 km^2^, 4,157,000 inhabitants and characterized by approximately 50% of populations being ethnic minorities (e.g., Dai). For simplification Mangshi county level city is further named as “county” in this manuscript. The altitude is approximately 900 m above sea level with a sub-tropical monsoon climate. Dairy ruminants are mainly water buffalo.

In Yunnan, farming systems for dairy cattle, goats and buffaloes consist mainly of backyard farms, dairy cooperatives and few commercial farms. Backyard farms are attached to a household and keep usually less than 20 ruminants, during the time of our survey less than 10. Cooperative farms consist of groups of backyard farmers which were encouraged and supported by the government to join a farm cooperative. They vary widely by size across China. In the study area and at the time of the survey these types of farms consisted of 10–20 individual farms. Those farms share some equipment but remain separated business units. Commercial farms vary widely in terms of size across different provinces and regions in China and can be classified as small, medium and large, respectively. Farms with fewer than 500 cows are usually considered small, those with 500 to 1000 cows are medium, while those with over 1000 cows are large. Commercial farms enrolled in this study can be classified as small or medium.

### 2.2. Data Collection

**Figure 2:**
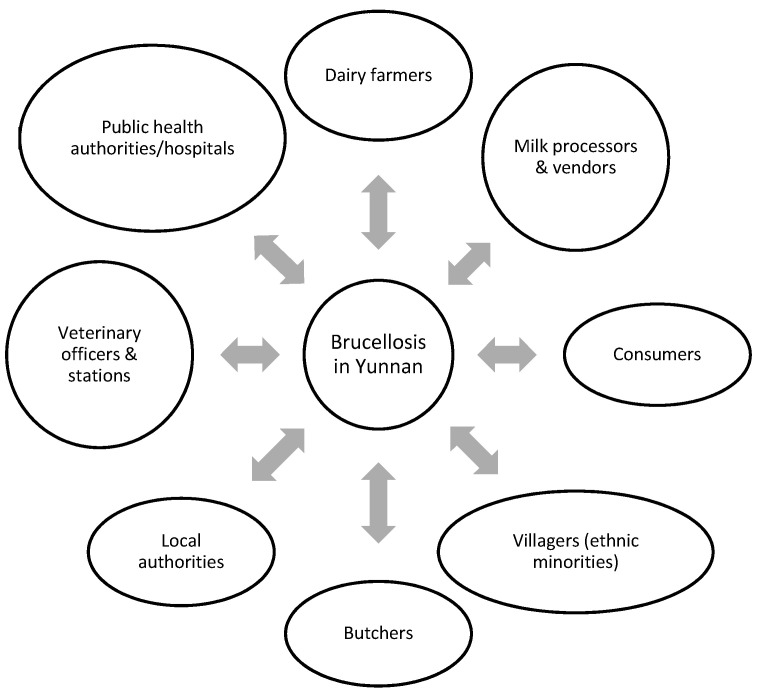
Stakeholders identified and included in the participatory study design.

An Ecohealth framework was developed indicating which stakeholders/groups should be included and which survey tools should be used (Figure 2). Quantitative data was collected using on-farm questionnaires and focused on production system, productive disorders and knowledge on zoonoses. All data collection tools were developed jointly with inputs from researchers of the four participating institutions, providing expertise on animal production, veterinary and public health. The questionnaire used is included in Supplementary Material S1. This was followed by a critical review and pre-test in the field before implementation. Special training was provided to the investigator but also to enumerators by a social scientist and a field epidemiologist on the use of the tools.

A multi-stage sampling design was applied within each county. Following this, two townships, and within each township two villages, were selected purposively based on dairy animal population data. One to two commercial farms were selected in each county with up to four workers interviewed in each. In addition, 1–2 cooperative farms were chosen in each township, with more than one farmer interviewed in each. Those farms were selected purposively. Within each village up to 10 backyard farmers were selected randomly for the interviews from an existing list of active backyard farmers. Farmers who did not accept to be enrolled were replaced (n = 8). Overall, 192 farmers were included (Table 1). The sample size calculation for backyard farms followed the assumption of an expected prevalence of 3.3% based on a historical report from Shilin county (12) and a margin error of 2.5%; cluster effects were not considered. Data was also collected qualitatively using focus group discussions (FGD), where villagers with and without ruminants were assigned to different groups; workers from commercial farms formed another group. Villagers without ruminants were included to evaluate their awareness on zoonoses, in comparison to those with ruminants. Even though most villagers have history of ruminant raising they were classed as “without ruminants” if they did not have them at the time of the FGD.

We also conducted in-depth interviews (IDI) for village animal health workers, butchers and doctors, focusing on knowledge, perception and awareness on zoonoses.

To further understand the disease burden of brucellosis in both humans and livestock, a retrospective investigation was carried out in hospitals and veterinary stations in the two project sites, and hospital outpatient records were reviewed. This included suspected cases of brucellosis using an accepted case definition for brucellosis-like symptoms in humans. This included hospital records of patients with recurrent fever of unknown origin, arthralgia, and headache. A total of 4 hospitals (2 in each county), and 6 veterinary stations (2 township and 1 county level from each county) were included in the study.

### 2.3. Serological Screening

Farmers, workers at a milk processing unit, village animal health workers and doctors were asked to volunteer a blood sample for serological analysis. The provincial Center of Disease Control (CDC) had the required ethical permits to perform this human sampling, and farmers were informed of the purpose and that participation was voluntary. Farmers refusing blood samples were still allowed to participate in the rest of the survey. Serum samples were tested for the presence of brucella antibodies using a serum (tube) agglutination test (SAT) and standard antigen, positive and negative control serums were from China CDC. The SAT was performed according to the national health industry standard for brucella diagnosis (WS268-2007) with a titer of 1:100, considered positive.

To get information on the situation of brucellosis among animals, bulk milk samples, which consisted of pooled milk from all the milking animals (buffalo, goat and cattle), were collected from all farms interviewed with the questionnaire. At each farm, a bulk milk sample (5 mL) was collected, except in cooperatives when all animals, even from different owners, were considered as one epidemiologic unit and only one sample was collected from the milk tank. The bulk milk samples were tested using an indirect ELISA kit (BRUCELISA-160M, Animal Health and Veterinary Laboratories Agency, UK, batch No. M01) for detection of antibodies against *Brucella abortus*. Laboratory tests and result interpretation was performed according to the instructions of the kit producer. Screening of brucella antibodies in milk samples were done by the Yunnan Animal Science and Veterinary Institute.

### 2.4. Ethical Approval Information

Verbal consent was obtained from each respondent attending the questionnaire, followed by focus group discussions and in-depth interview, also from dairy cow, buffalo and goat owners during collection of milk and human serum samples. Sampling and serological screening procedures performed within this study followed the national standards for surveillance of brucellosis in humans and animals, and approved by ethical committees of the Yunnan Agricultural University (clearance code: 2012-59) and Yunnan Institute of Endemic Disease Control and Prevention (clearance code: 2011-10).

### 2.5. Data Analyses

Questionnaires were analyzed descriptively. To determine association between county and demographics, a Chi-square test was used, as well as logistic regression with towns and villages as random effects (STATA command meqrlogit). For the association between farm type and previous abortions, one-way ANOVA with Bonferroni correction was used, and as this question was only answered in one county, it was not relevant to include county as a random effect. For data collected qualitatively, content analysis was used. Triangulation was used to compare quantitative and qualitative data.

## 3. Results

### 3.1. Demographic Data from the Questionnaire

In total, 192 questionnaires were administered from 200 interviewees; 8 of them did not consent (response rate 96%). In total, 74% of the respondents were male and 26.1% female. In Yiliang significantly (*p* < 0.001), more respondents were women (37.9%) compared to Mangshi (4.4%). The age of interviewees did not differ between both regions, with an average age of 45 years (std = 0.9) for Yiliang and 36 (std = 1.25) for Mangshi, respectively. Significant differences (*p* < 0.001) were found in ethnic group of interviewees between the study areas, with 68.6% of farmers in Yiliang being Han and 67.6% in Mangshi being Dai, and only 30.9% Han. The majority of the enrolled dairy buffalo farmers in Mangshi were Dai (88%). There was a high proportion of participants with no higher education beyond primary school (39.7% in Mangshi and 49.2% in Yiliang).

### 3.2. Information on Disease Priorities, Management, and Risk Factors

In total, 17 FGD were conducted with 123 participants. Among these, 7 FGD in Yiliang (3 groups for farmers with ruminants, 3 groups for farmers without ruminants, 1 group for commercial farm) with 49 participants; and 10 FGD in Mangshi (4 groups for farmers with ruminants, 4 groups for farmers without ruminants, 2 groups for commercial farms) with 74 participants, were carried out. Analysis of the questionnaire, FGD and IDI results found that both regions considered Foot and Mouth Disease (FMD) as the most important disease, followed by respiratory diseases or related symptoms. The targeted zoonosis (brucellosis) was not considered as important. However, one of the leading symptoms for brucellosis (abortions) was mentioned as the 3rd most important disease symptom in ruminants for farmers in Mangshi.

Results from the questionnaire revealed that abortion was very common. The question if cows had aborted during the last 12 months was only answered in Mangshi, where 83.1% of dairy farmers had faced bovine abortion over the last 12 months (100% of cooperatives and commercial farms). Further, 65–80% of respondents stated that they handle abortion cases in cattle by themselves without consulting a veterinarian. Farmers in all three farm types stated that they were most likely to keep cattle with an abortion history and inseminate again (Table 2). More than 50% of respondents indicated that they still sell or use the milk for home consumption produced after the next successful parturition from cattle with an abortion history.

Disinfection of the cattle barn and surroundings as a disease prevention measure was used by almost all respondents. Only one respondent did not apply this at all. Frequency of disinfection of barns and surroundings varied by region. It was found that 57.7% applied it at least bi-weekly in Mangshi, while only 89.2% did in Yiliang. Overall, around half of respondents used quarantine measures (separation and increased disinfection) when introducing animals, but there was significantly more in Yiliang that did this (Table 3). When respondents who did not apply quarantine measures were further asked their reason, 40% of them stated that this measure is not considered to be important. Milk equipment sharing was common in cooperative farms, but not in backyard farms.

Veterinary services were found easily accessible also by FGD participants. Cross checking with questionnaires, the majority of veterinarians (certified veterinarians or village animal health workers) were from the same township, village or neighboring village, with more than 50% from the same or neighboring village (Table 3). When accounting for the random effects of village and town, there was no significant difference between the counties.

### 3.3. Knowledge, Perception and Awareness on Zoonoses

Knowledge of potential ways of transmission for brucellosis was poor across both regions and all three farm types, with at least 71% of respondents from the questionnaire unable to respond to this question. The limited knowledge of farmers on zoonoses observed in on-farm questionnaires was also prominent in the FGD. If any zoonoses were known to participants of FGD it was always related to other diseases than brucellosis e.g., rabies, FMD, avian influenza, bovine spongiform encephalopathy (BSE), cysticercosis or tuberculosis (TB). No difference in zoonoses knowledge between villagers with/without ruminants were observed.

Exchange between farmers and watching TV was seen as the main channel of the villagers’ access to information. However, for minorities in Mangshi, watching TV might be difficult because of the language barrier, and they may be lacking relevant information because of this.

Results from the IDI demonstrated also that the three professional groups, namely village animal health workers, village doctors and butchers in both study areas had low awareness of zoonotic diseases. By comparison, butchers had the lowest awareness of zoonotic diseases among the three groups. The most commonly mentioned zoonotic diseases were FMD, rabies, and avian influenza. Only two animal health workers out of 17 mentioned brucellosis as a zoonosis and thought it was an important but not common disease.

“I did not hear about brucellosis, I might learn them when studying in school, but I forgot all about them.”—A village animal health worker in Mangshi, male, Dai ethnic minority group, 39 years old.

Lack of diagnostic criteria and laboratory tests for brucellosis and other zoonotic diseases was evident at village and township levels of both veterinary and health systems. Village animal health workers and doctors stated that there is only little communication and collaboration between health and veterinarian departments, and joint action only in urgent matters.

### 3.4. Review of Hospital Cases and Vet Station Data

Through retrospective investigation in the public health sector in the two project sites, 56,883 outpatient medical records were checked, 1103 cases of febrile illness were found, but in only 341 of them were further diagnostic tests carried out to identify the cause. Zero cases were confirmed as brucellosis. Likewise, no cases were found in the survey of records of 6 veterinary stations.

### 3.5. Results from Serological Survey in Farms (Bulk Milk) and Human

The testing of bulk milk samples from Yiliang (N = 85) found 5 positives out of 45 samples from dairy cattle (11.1%, 95% confidence interval (CI) 3.7–24.1%) and one in 40 dairy goat samples (2.5%, 95% CI 0.1–13.2%) (Table 3). Testing of human sera found that 5 samples out of 126 (3.97%, 95% CI 1.3–9.0%) samples from Yiliang were serologically positive for brucellosis. Titers of positive tested humans ranged from 1:100 to 1:3200. Of these, 4 positive samples were from one cooperative dairy cattle farm (3 owners and 1 worker), and 1 farmer of a backyard dairy goat farm. In Mangshi, all 102 human serum samples and 41 bulk milk samples were negative for antibodies against *Brucella abortus* (Table 4). Collected quantitative data shows a history of abortion (number varying from 3–30) in this cooperative. In two of the positive dairy farms there were no reports of previous abortions. The two farms where humans were seropositive had both experienced abortions, but in the dairy goat farm, the milk sample was negative (Table 5).

## 4. Discussion

This study found that both ruminants and ‘humans in contact with livestock’ had been exposed to brucellosis in Yiliang but not Mangshi counties of Yunnan, even though the serological results do not prove on-going nor recent transmission.

Brucellosis prevalence in goats in our study was 2.5% for Yiliang. While livestock data on brucellosis is scarce (or not up to date) for China, including Yunnan, a report from 1998 indicated a prevalence in dairy goats in Shilin County, neighboring Yiliang, of 3.3% (goats) and 25.8% at farm level, and two *B.melitensis* strains were isolated [12]. In our study, positive samples were found both in milk samples of goat and owner’s serum samples in a goat farm.

Serological results for ruminants using milk ELISA need to be interpreted with caution due to sensitivity and specificity issues. We used the BRUCELISA 160M in our survey. The ELISA was performed according to manufacturer’s guidelines. Details on Sensitivity (SE) and Specificity (SP) were not provided by the supplier at the time of the study. However, the Yunnan Animal Science and Veterinary Institute had conducted some in-house evaluation related to SE and SP, and recommended to use the test. While milk ELISA is not perfect, it is one of the most reliable and cost-effective tests for brucella antibodies that can be performed on milk [6,13].

Data on prevalence of brucellosis in human has been recorded since 1989: Surveillance of brucellosis in 111 counties, covering 50,366 farmers, butchers, vets and workers from 1990 to 1996 confirmed 591 positive in 64 counties, with a positive rate of 1.17%, and 168 cases being recorded [14]. Our study conducted human sampling in specific risk categories, and thus the results are not comparable. Still, with 5 out of 126 volunteers being serologically positive for brucellosis in Yiliang country, the potential risk for transmission to humans was demonstrated. This is in line with a recent long-term analysis (1950–2018) of brucellosis in humans in China (including Yunnan) which indicated for 2004–2018 the most significant increase in incidence included Yunnan among other provinces [8]. No confirmed cases were found in hospitals in the two project sites, which may reflect the under-reporting by local doctors due to limited diagnostic capacity; cases have been diagnosed elsewhere, e.g., in the First Affiliated Hospital of Kunming Medical University, where 28 confirmed cases were treated from January 2008 to April 2014 [7].

Demographic data from the questionnaire found that in Yiliang County more respondents were women compared to Mangshi. Women in Yiliang may be more likely to be involved in livestock keeping in Yiliang, since it is easier for men to find jobs in Kunming, and therefore women remain behind.

In this study, abortion, the leading symptom for brucellosis in livestock, was found to be very common in bovines. Considering that there is lack of a clear diagnostic criteria and laboratory testing for brucellosis at village and township levels of both veterinary and health systems, it is likely that the disease is under-reported. More and more backyard farmers are encouraged by the authorities to join cooperatives, with the possible gains of better cost sharing, feed and expected disease control. However, animals from different origins can pose a higher risk for any disease spread, and it has also been shown that larger herds are more likely to have seropositive animals than smaller herds, and organized farms are also at higher risk compared to backyard farming [15]. It is therefore increasingly necessary to develop better strategies for brucellosis prevention and control in cooperative farms.

### 4.1. Limited Knowledge, Awareness and Perception Hamper Effective Control of Zoonotic Disease

Prevention and control strategies of brucellosis include identifying and eliminating infected animals with brucellosis, vaccination programs aimed at reducing the prevalence of disease in livestock, decreasing occupational exposures, and establishing surveillance, particularly in districts with cases of brucellosis (clinical outbreaks or serological positive animals) over the last few years. Also, increasing local knowledge of appropriate food handling techniques of dairy products including pasteurization are crucial [16]. This study found a general low knowledge and perception on zoonoses and brucellosis in respondents elucidated through FGD, IDI and questionnaires. People were generally not aware about brucellosis, but could name other zoonoses, including rabies and tuberculosis. Interestingly, FMD was also mentioned as a zoonosis, and while this is technically true and FMD can infect humans [17], this is very uncommon and a negligible risk, and the answers here are most likely reflecting misperceptions, such as the common confusion with ‘hand, foot and mouth’ disease in humans (non-zoonotic). We found also poor knowledge on brucellosis in village animal health workers. This might be explained by the fact that they received only short training courses on specific tasks, e.g., vaccinations and often lack formal education. Low knowledge about brucellosis has been found in other countries, e.g., India and Tajikistan [18,19], which ironically leads to the disease frequently being neglected.

Though abortions were common and are one of the leading symptoms for brucellosis on all farms, farmers do not consider brucellosis as a problem, likely because of low knowledge and more emphasis being put on other diseases, such as FMD and avian influenza. Risk factors, such as keeping and milking of animals with abortion history, insufficient quarantine measures implemented to new introduced animals, and the sharing of milk equipment, in particular common practice at cooperative farms, are all likely to contribute to further spread. Studies have shown the strong link between abortions and brucellosis [6]; to reduce these risk factors it is suggested that local authorities should put emphasis on education and training for cooperative farms. A recent analysis of the effects of control programs for brucellosis in China found that there seems to be a re-emergence of the disease, particularly in the northern provinces, and increasing vaccination campaigns with elimination of infected animals would be necessary to reduce the trends again [11].

### 4.2. Use of the Transdisciplinary Approach Achieved Added Value

There is only limited communication and collaboration between public health and veterinary systems, with the exception of major disease epidemics such as avian influenza, when they work together in the control of the epidemic. Therefore, the collaboration is reactive but not proactive and short term, rather than routine, long term and sustainable. Through the implementation of the Ecohealth approach, we demonstrated throughout that it is possible for different disciplines to work successfully together from implementation until analysis. Introduction of qualitative tools such as FGD or IDI was well received by a team which had never used it before, but gained capacity and appreciated their added value.

All the activities in field survey for this Ecohealth project provided a good opportunity for team members with different background of animal production, animal health, public health, high education and social science to target an integrative approach to improve knowledge, better health and better development. For most team members, it was the first time for them to work together to do activities for the same research project.

### 4.3. Further Ways of Influencing Authorities to Understand, Accept, and Use the Ecohealth Approach

Rapid economic growth within the region is linked to and driving major changes in ecosystems and social systems which create conditions ideal for the emergence and spread of new diseases. The H5N1 epidemic demonstrated the need for improved collaboration between sectors and stakeholders. Ecohealth and One Health (OH) have been recently introduced to Southeast Asia to address these needs [20]. The idea behind them is to move away from silo thinking towards the involvement of various disciplines such as socioeconomic, environmental, social-science, ecological and bio-medical expertise [21]. Charron [15] stated in relation to the use of Ecohealth that decision-makers and communities should be involved in guiding research priorities and acting on conclusions and recommendations from such research. Additionally, it has been common practice that many projects are driven by priorities of northern countries, rather than lower-income countries which have a high burden of infectious diseases and differing priorities. However, classical control measures proved to work in developed countries (e.g., culling) may fail in poor resource environments. Current control of EID in China is rather top-down driven and collaboration across different disciplines is often not established. Most affected groups, such as farmers, are often excluded from any decision process. To further educate academics, a teaching course to introduce Ecohealth or One Health to veterinary public health masters’ students in Yunnan Agricultural University has been launched. For the public health authorities, a China national anthrax prevention and control guide was being prepared using participatory principles with Yunnan Institute of Endemic Disease Control and Prevention. The provincial level is adding modules on zoonoses in their ongoing health education.

### 4.4. Limitations

The authors acknowledge some limitations of the study. Certain farm types were selected purposively such as commercial and cooperative farms. This could lead to a selection bias. To minimize this bias, the selection of those farms was done in a consultative process between the various team members and involved authorities and groups. In addition, cluster effects were not considered in the sample size calculation for backyard farms, mainly due to budget limitations. While this reduces the statistical power to demonstrate difference, the research team decided to go ahead to allow the team to provide first-hand information in absence of other data. The review of available literature to date has shown that information on brucellosis is still scarce. FGD and IDI were limited in numbers and may not have been done until full saturation. While this may pose a risk that not all opinions and beliefs of participants have been captured, the research team feels confident that the main issues have been explored and understood sufficiently. The Ecohealth approach had not been applied before in the area, and a lot of capacity building was needed to facilitate the qualitative surveys and the transdisciplinary work. It is suggested that future surveys should try to minimize the described limitations and learn from the lessons of this project.

## Figures and Tables

**Table 1:** Summary of data collection done in two counties in Yunnan Province, China.

Tool/Method	Targeted Group/Actor	Mangshi *	Yiliang	Total
Focus group discussions	Villagers with ruminants	4	3	7
	Villagers without ruminants	4	3	7
	Commercial farm	2	1	3
In-depth interviews	Village animal health workers	10	7	17
	Local butchers	10	4	14
	Village doctors	10	6	16
Questionnaire	Dairy farmers	68	124	192
Retrospective investigation	Hospitals	2	2	4
	Veterinary stations	3	3	6

* County level city.

**Table 2:** Abortion (last 12 months) and handling in different farms in two counties in Mangshi county, Yunnan province, China.

Farm Type	Abortion History	Retain Cattle with an Abortion History and Inseminate Again
Backyard (N = 31)	67.7% *	94.0%
Cooperative farms (N = 25)	100% *	90.0%
Commercial farm (N = 3)	100%	83.3%

* Significantly different at *p* = 0.003 from cooperative farms with one-way ANOVA.

**Table 3:** Management factors and access to vet service in two counties in Yunnan province, China. Data from questionnaire.

Brucellosis Prevention	Overall	Mangshi (N = 124)	Yiliang (N = 68)	*p*-Value *
Disinfection of the cattle barn and surroundings more than twice per month (N = 188)	129/188 (68.6%)	71/123 (57.7%)	58/65 (89.2%)	<0.001
Quarantine measures (N = 184)	101/184 (54.9%)	41/117 (35.0%)	60/67 (90.0%)	<0.001
Keep cows with abortion history for breeding (N = 146)	135/146 (92.5%)	95/104 (91.4%)	40/42 (95.3%)	0.4
Veterinary services available in the same or neighboring village (N = 191)	90/191 (47.1%)	75/123 (61.0%)	26/68 (52.9%)	0.003

* Chi2 test.

**Table 4:** Milk and serum samples tested for Brucella antibodies in Yunnan Province, China.

Samples	Targeted Group/Actor	Mangshi		Yiliang	
		Sample Size	No. of Positive	Sample Size	No. of Positive
Animals	Commercial farms				
(Bulk milk)	Dairy cow	0	0	1	0
	Dairy buffalo	1	0	0	0
	Backyard farms				
	Dairy cow	0	0	39	3
	Dairy buffalo	36	0	0	0
	Dairy goats	0	0	40	1
	Cooperative farms				
	Dairy cow	0	0	5	2
	Dairy buffalo	4	0	0	0
	**Total ruminants**	**41**	**0**	**85**	**6**
Human (serum)	Animal health workers	10	0	7	0
	Public health workers	10	0	9	0
	Farmers *	70	0	104	5
	Others **	12	0	6	0
	**Total humans**	**102**	**0**	**126**	**5**

* Including owners and farm employees; ** Butchers and milk processors.

**Table 5:** Abortion history at farms with either seropositivity in either cattle or humans in Yiliang County, Yunnan province, China.

Farm Type	Bulk Milk Samples	Human Serum Samples	History of Abortion on Farm	Sum of Abortion *
Dairy cow/cooperation	Positive	1 employee and 3 cattle owners positive	Yes	3-30
	Positive	Negative	Yes	5
Dairy cow/Backyard	Positive	Negative	No	-
	Positive	Negative	Yes	2
	Positive	Negative	No	-
Dairy goat/Backyard	Positive	Negative	Yes	20
	Negative	1 farmer positive	Yes	3

* Reported number of abortions last year by farmer. At cooperatives, the different farmers could give different answers.

## Data Availability

Data will be made available from the authors on request.

## Supplementary Materials

The following are available online at https://www.mdpi.com/article/10.3390/tropicalmed6030134/s1, Questionnaire S1: Farm questionnaire.
