# Supplementary material for: Participatory and Transdisciplinary Studies of Brucella Infection in Humans and Animals in Yunnan Province, China—Lessons Learned
Source: Trop Med Infect Dis. 2021 Jul 15;6(3):134. doi: 10.3390/tropicalmed6030134 (PMC8293356; doi:10.3390/tropicalmed6030134)
Supplement: Supplementary file 1 [file tropicalmed-06-00134-s001.zip › tropicalmed-1224069-supplementary.pdf]

On-farm/household, questionnaire

Applied to: goat farms & cooperative dairy herds (buffalo and cattle)

Personal questionnaire

Questionnaire Numbers: Serum/Milk Collection Numbers:
Surveyor Name:
Survey Date Year Month Day
Survey Site County Townships Village
Surveyor

Whether By respondents I answer the questionnaire or not ( ) ①Yes ②No

1. General Personal Data

- Name of respondent: ( )
- Respondent ( ): ① owner ② manager ③ worker ④ family member
- Gender ( ) ① Male ② Female
- Age ( )Years old
- Nationality ( ) ① Han ② Yi ③ Dai ④ Others (specify:)
- Educational level ( )
  ① Primary ② middle ③ high school ④ university/collage
  ⑤ other (specify:)
- How long in business ( ) years
- Number of family members: ( ) man, ( ) woman ( ) children(<16)
  How many of them involved in livestock business: ( ), man ( ) woman
- No of hired workers currently involved in livestock business:
  ( )man, ( ) woman

2. Production data/socio economic

- Animal species/breed, numbers (   )

Species	Target purpose	Total No	Breed 1 (specify)	Breed 2 (specify)
Cattle				
Goats				
Buffalo				
Pigs				
Others				

- Farm type (   )

  ① cooperative   ② small holder   ③other (specify:)

- Production figures
  - How many calves/lambs born (   ) per year
  - Milk yield at average (   ) per day
  - How many bulls/goats for sell (   ) per year

- Entry of animals (last 12 months)

Species	Total	Same village	Same District	Other (specify)

If it is from the own reproduction, the township and village are (   )

- Exit of animals (last 12 months) (township)

Species	Total	Same village	Same District	Other (specify)

- What do you do with the milk (　　　　　)

① own consumption ② sell ③ make cheese (own processing) ④ other

- Where is the produced milk supplied to? (　　)

① same village ② same district ③ other district

- Do you supply milk to a dairy company or milk vendor: (　) ① yes ② no

  If yes, provide address: (　　　　　　　　　　)

- Land use (　　) ①own ②rented ③purchased
- Feeding with（　　）

  ①crop ②corn ③grass and silage ④other(specify:　　)

- Income
  - Total income (Yuan) in last 12 months
  - Income (Yuan) by category in last 12 months

domestic livestock: (　　　) from crops, (　　　) from both animals, and crops(　　　) from others (specify:　　　　　　　　　　)

  - Income trend 2010 and 2011: (　) ① upwards ② downwards

If change observed, what is the most important reason: (　　　　　　　　　　)

3. Animal health

- List observed animal diseases or symptoms in the last 12 months

Species	No of animals in stock	No of animals infected	No of Death	Disease/symptoms

- Ranking of the first 3 most important

Disease/ symptoms	Loss from the disease	Action to be undertaken	Rank by importance

4. Disease prevention

- Access to Vet Services

What do you do when you find a sick animal? By (   ) ① company vet ② local vet ③ treat on my own ④ others (specify:)

- Vaccination

Type of vaccine	From where	When applied	By whom	Performance

- Does a farm gate bio-security measure exist? ( ) ① yes ② no

If not, why not ( ) ① not important ② not comfortable ③ too expensive

- (4) How often is the measure of animal house cleaning performed?

Please specify: ( )

- Is there a disinfection program performed in farm? ( ) ① yes ② no

If yes, please specify: (

)

- If there is a sick animal, what do you do with it? ( ) ① Destroy ② keep it in separation ③ keep it without separation ④ sell ⑤ other (specify:)

- If you have a news that a disease is occurring in your township or village, what do you do with your animal farm? Please specify: (

)

- What do you do with a dead animal body? ( )

① sell for slaughter ② meat for family consumption ③ deep bury with disinfection ④ others
(specify)

- Quarantine for newly-introduced animals,

If you introduce a new animal, do you have a quarantine measure? ( )
① yes ② no
If yes, please specify how: ( ) and why: ( )
① avoid disease ② follow regulations ③ advice from vet ④ other
If not, why not: ( )
① not important ② not comfortable ③ too expensive ④ other

- If a newly-introduced animal is sick, what do you do with it? (   )

  ① keep it in separation ② keep it without separation ③ give it back to the former owner ④ report to officers ⑤ other
- When you have a plan to introduce animals, what do you most concern?

  ① breed ② price ③ disease ④ other
- When you introduce animals, do you ask for an animal health certificate?

  ① yes ② no
  - If yes, which disease are of your concern

  If no, why not? Please specify: (   )
- Do you share pasture (communal grazing) or equipment (e.g. for milking) with other farmers/villagers ① yes ② no

5. Reproductive disorders

- Survey of abortion history (over last 12 months)

Type	No	When (month)	Status of pregnancy			Comments
			early	mid	late	
Heifer						
Cow						
Goat (1st pregnancy)						
Goat (>2nd pregnancy)						

- How does the fetus look like: (   )

  ① fresh ② leathery ③ rotten ④ unknown

- What are the possible cause of the abortion: (   )

  ① trauma ② new animals introduced ③ other (specify:)

④ unknown

- What do you do with the aborted materials? (   )

  ① deep burying ② feed to dog ③ others (specify:)

  ④ unknown

- If ever, did you use gloves when you handled the abortion materials/fetus? (   ) ① yes ② no

  if no, why not? (   ) ① not comfortable ② too expensive ③ not needed

- How do handle the problem of observed abortion in animals? (   )

  ① inform vet ② handle on my own

- What they do with the female with abortion? (   )

① still keep it ② inseminate it again ③ slaughter for meat ④ sell it

⑤ others (specify:)

- What do you do with the milk from a cow with an abortion history? (   )

① still milking ② milking, but not use for human consumption ③ discharge
④ others(specify:)

6. Infertile cows/goats/buffalo (adjust by species)

- Herd structure (add goats)

Total number of cows/goats	Calves	Heifers	Bulls

- Herd fertility (cows)(adjust by species)

Total no. cows/goats	Pregnant	Not pregnant for 12 months	Not pregnant for 24 months

- What do you do with cows which do not give birth in the last 2 years?(adjust for goats)

  ①still keep ②sell ③other

- Herd fertility (bull)

What do you use for breeding purpose?

① bull (own) ②bull (neighbor) ③AI

④other (specify:)

- Bull not successfully breeding performance (what they do with such a bull/goat): (specify:)

7. Zoonoses

- Do you know any disease which can be transmitted from animals to human?

  ( ) ① yes ② no
  If yes, please kindly fill the table below

Serial	Name of diseases	Symptoms	Mode of transmission
1			
2			
3			
4			
5			

- Do you have any specific knowledge for Toxoplasmosis and Brucellosis?

  ( ) ① yes ② no

if yes, please kindly fill the table below

Name of diseases	Symptoms	Mode of transmission
Brucellosis		
Toxo		

- What are the potential risk factors for transmission of Brucellosis? (   )

  ① contact to sick animal ② drink milk ③ eat meat ④ handle either milk or meat ⑤ keep pet animals

- What are the potential risk factors for transmission of Toxoplasmosis?

(   ) ① contact to sick animal ② drink milk ③ eat meat ④ handle either milk or meat ⑤ keep pet animals

- What are the key factors when handling milk for self consumption? (   )

① drink fresh milk ② drink less ③ cook well ④ keep in icebox ⑤ keep in room temperature

8. Zoonoses Human(Case survey)

- What symptom do you endurance?
  - fever （　　） ①yes ②no

    If yes, fever lasted for （　　） days, the temperature was (　　)℃
  - sweating （　　） ①yes ②no
  - joint & muscular pain （　　） ①yes ②no
  - testes inflammation （　　） ①yes ②no

- Livestock contact history (tick the relevant one)
  - pasture cattle or goats（　　）

- butcher （ ）
- artificial insemination （ ）
- vet （ ）
- skin lamb with bare hands （ ）
- other (specify:)

- Possible transmitted route （tick the relevant one）
  - occupational contact with infected animals （ ）
  - ingesting raw milk （ ）
  - ingesting cheese （ ）
  - contact with *Brucellosis* patients （ ）

9. Environment

- Transportation:(Km) from main roads, (Km) from village, (Km) from the nearest farm.
- Water supply from( ) ① well ② tape water ③ a shared river ④ rainwater
- Does the office, production area and workers living area separated? ( ) ① yes ② no
- Does there is a waste-processing areas in your farm? ( )

  ① yes ② no
- How do you deal with the manure? ( )

  ① sell to organic fertilizer company ② self use (manure to own land) ③ discard ④ other (specify:)
